# Indoleamine 2, 3-Dioxygenase 1 Mediates Survival Signals in Chronic Lymphocytic Leukemia *via* Kynurenine/Aryl Hydrocarbon Receptor-Mediated MCL1 Modulation

**DOI:** 10.3389/fimmu.2022.832263

**Published:** 2022-03-18

**Authors:** Claudio Giacinto Atene, Stefania Fiorcari, Nicolò Mesini, Silvia Alboni, Silvia Martinelli, Monica Maccaferri, Giovanna Leonardi, Leonardo Potenza, Mario Luppi, Rossana Maffei, Roberto Marasca

**Affiliations:** ^1^ Hematology Section, Department of Medical and Surgical Sciences, University of Modena and Reggio Emilia, Modena, Italy; ^2^ Center for Neuroscience and Neurotechnology, University of Modena and Reggio Emilia, Modena, Italy; ^3^ Department of Life Sciences, University of Modena and Reggio Emilia, Modena, Italy; ^4^ Hematology Section, Policlinico, Department of Oncology and Hematology, Azienda Ospedaliero-Universitaria (A.O.U.) of Modena, Modena, Italy

**Keywords:** chronic lymphocytic leukemia (CLL), tumor microenvironment, indoleamine 2, 3-dioxygenase 1, target therapy, aryl hydrocarbon receptor (AHR)

## Abstract

The indoleamine 2,3-dioxygenase 1 (IDO1) metabolic circuitry, comprising the first tryptophan (Trp) catabolite L-kynurenine (Kyn) and the aryl hydrocarbon receptor (AHR), has emerged as a mechanism of cancer immune evasion. Here, we investigated the functional role of the IDO1/Kyn/AHR axis in chronic lymphocytic leukemia (CLL). Our data show that CLL cells expressed an active form of the IDO1 enzyme and microenvironmental stimuli can positively modulate its expression. Interferon (IFN)-γ induces IDO1 expression through the Jak/STAT1 pathway and mediates Kyn production concomitantly with Trp consumption in CLL-conditioned media, while INCB018424 (ruxolitinib), a JAK1/2 inhibitor, impaired both effects. To characterize the involvement of IDO1 in leukemic cell maintenance, we overexpressed IDO1 by vector transfection measuring enhanced resistance to spontaneous apoptosis. IDO1 pro-survival influence was confirmed by treating CLL cells with Kyn, which mediated the increase of induced myeloid leukemia cell differentiation protein (MCL1). Conversely, AHR silencing or its blockade *via* CH-223191 improved the apoptosis of leukemic clones and mitigated MCL1 expression. Moreover, Kyn-treated CLL cells are less affected by the pro-apoptotic effect of ABT-199 (venetoclax), while CH-223191 showed synergistic/additive cytotoxicity with this drug. Lastly, targeting directly MCL1 in CLL cells with AMG-176, we abrogate the pro-survival effect of Kyn. In conclusion, our data identify IDO1/Kyn/AHR signaling as a new therapeutic target for CLL, describing for the first time its role in CLL pathobiology.

## Introduction

Chronic lymphocytic leukemia (CLL) is the most common leukemia in the western world, usually affecting the elderly (1). CLL is a dynamic disease in which proliferation of the antigen-experienced B cell clone in pseudofollicular centers of lymphoid tissues is combined with a reduced cell turnover of quiescent B cells that accumulate in peripheral blood (2). The survival of leukemic cells is closely linked to the presence of a specific tumor microenvironment (TME). Malignant cells are able to manipulate surrounding supportive cells by forcing them to create a niche in which they can receive survival stimuli, escape immunosurveillance, and protect themselves from the action of drugs (3). Moreover, CLL cells play an active role in establishing progressive immunosuppression, demonstrated by the presence of an expanded population of regulatory and exhausted T cells, myeloid-derived suppressor cells (MDSCs), and macrophages with the M2 phenotype, called nurse-like cells (NLCs). Several treatments available for CLL exert a long-term control of the disease in the majority of CLL patients, as novel tyrosine kinase inhibitors and the B-cell lymphoma 2 (BCL2) inhibitor ABT-199 (venetoclax) (4), but CLL still remains largely incurable due to frequent relapses and the emergence of drug resistance or intolerance (5).

Indoleamine 2,3-dioxygenase 1 (IDO1) is the rate-limiting and first enzyme of the kynurenine pathway that converts the essential amino acid L-tryptophan (Trp) to produce bioactive metabolites in mammalian extrahepatic tissues (6). Since its discovery, IDO1 was described as an immunomodulatory molecule, able to promote immune tolerance in mammalian pregnancy (7), chronic infection, autoimmunity, and allergic inflammation (8). Inflammatory mediators, in particular interferon (IFN)-γ (9), induce and sustain IDO1 production in a variety of cells as a feedback mechanism to control inflammation and dampen overactivation of cytotoxic T responses (10). The first Trp catabolite, L-kynurenine (Kyn), is a key signaling molecule that directly affects antigen-specific T-cell proliferation (11, 12) and induces T-cell death indirectly through the aryl hydrocarbon receptor (AHR), also known as the dioxin receptor (13, 14). IDO1 is overexpressed in a wide variety of human hematologic malignancies and solid tumors as a part of concerted mechanisms of evading immunosurveillance (15–17). In addition, many cells belonging to the TME—fibroblasts, macrophages, MDSCs, and dendritic cells, including endothelial cells—are coerced by cancer cells to express IDO1, collectively supporting the immune escape (12). Besides, IDO1 expression is correlated with a poor prognosis, shorter overall survival, and chemoresistance in different cancers (18–24), corroborating the concept that a treatment strategy of IDO1 blockade may have antitumor effects. To date, a large number of IDO1 inhibitors have been reported, some of them including epacadostat, BMS986205, and indoximod, have advanced into clinical trials for cancer treatment (25).

In CLL, the activity of IDO1, measured as the plasma kynurenine-to-tryptophan ratio ([Kyn]/[Trp]), is reported to be increased (26). The expression of IDO1 is reduced in peripheral blood mononuclear cells (26), while NLCs (27) and CD14^+^HLA-DR^lo^ MDSCs (28) expressed high levels of this catabolic enzyme, and IDO1 inhibition may restore T cell proliferation. Even in studies on Eµ-TCL1 mice with a CLL-like disease, the enhanced expression of IDO1 in tumor-associated myeloid cells was observed (29). Interestingly, IDO1 expression was significantly higher in malignant CD19^+^ B cells of Eµ-TCL1 mice compared to CD19^+^ B cells of wild-type (WT) mice (29). However, the functional role of IDO1 in the leukemic clones from CLL patients is unknown. Here, we evaluated whether the IDO1/Kyn/AHR signaling pathway may be involved in CLL pathobiology. Our findings illustrate for the first time a role of the IDO1 metabolic pathway in CLL-prolonged survival and impaired drug sensitivity. The effects are mediated by the autocrine/paracrine action of Kyn that activates AHR. Interestingly, the blockade of AHR *via* CH-223191 interferes with the pro-survival signal and MCL1 induction triggered by IDO1. We also demonstrated that IDO1 metabolic activity affects the response to ABT-199, while CH-223191 treatment synergizes with the BCL2 inhibitor to kill leukemic CLL cells. Collectively, our findings suggest that the IDO1/Kyn/AHR axis may represent a novel therapeutic target in CLL.

## Materials and Methods

### Patients

Blood samples from untreated patients that matched standard diagnostic criteria for CLL were obtained from the Hematology Section of Modena Hospital, Italy, with a protocol approved by the Institutional Review Board. All patients provided written informed consent in accordance with the declaration of Helsinki. Peripheral blood mononuclear cells were isolated by density gradient centrifugation with Lymphoprep medium (Pharmacia LKB Biotechnology, Piscataway, NY, USA) and used fresh or cryopreserved in RPMI-1640 medium, 50% FBS, and 10% DMSO and stored in liquid nitrogen until use. To enrich for CLL cells, peripheral blood mononuclear cells were incubated with CD19-specific microbeads (Miltenyi Biotech, Auburn, CA, USA) and separated by autoMACS (Miltenyi Biotec).

### *In Vitro* CLL Stimulation and Drug Treatments

To analyze basal IDO1 levels, CD19^+^ CLL cells were serum starved for 1 h in RPMI-1640 at 37°C prior to RNA extraction. In all other experiments, CD19^+^ CLL cells were resuspended in RPMI-1640 medium + 10% FBS. To mimic microenvironmental stimulation, CLL cells were treated with one of the following soluble factors: IFN-γ 500 U/ml (PeproTech Cat# 300-02, Rocky Hill, NJ, USA); LPS 5 μg/ml (Sigma-Aldrich Cat# L5293, St. Louis, MO, USA); goat F(AB′)2 fragment to human IgM (5FCµ) 10 μg/ml (Thermo Fisher Scientific Cat# ICN55055, Waltham, MA, USA); Type B CpG oligonucleotides 1 μg/ml (ODN 2006) (InvivoGen Cat# tlrl-2006, San Diego, CA, USA); and CD40L 200 ng/ml + interleukin (IL)-4 20 ng/ml (both from PeproTech Cat# 310-02 and 200-04) or tumor necrosis factor (TNF)-α 5 ng/ml (PeproTech Cat# 300-01A). Control cells were cultured in parallel without stimulation. L-Kynurenine (Sigma-Aldrich Cat# K8625) was used at 100 μM. INCB018424 (ruxolitinib) (SelleckChem Cat# S1378, Houston, TX, USA) was used at 0.1 and 1 μM as previously described (30). CH-223191 (MedChemExpress Cat# HY-12684, Princeton, NJ, USA) was used at 10 μM. ABT-199 (venetoclax) (SelleckChem Cat# S8048) was used at 1 nM. AMG-176 (MedChemExpress Cat# HY-101565) was used at 100 or 300 nM.

### Real-Time PCR

Total RNA was extracted with the RNeasy Plus Mini Kit (Qiagen Cat# 74134) and reverse transcribed using SS VILO Master Mix (Life Technologies Cat# 11755050, Carlsbad, CA, USA). Twenty nanograms per reaction of cDNA was analyzed in real-time PCR on LightCycler 480 v.2 (Roche) using SYBR Green Master Mix (Applied Biosystems Cat# 4309155, Foster City, CA, USA). Specific primers designed for *IDO1*, *CYP1A1*, and *MCL1* are listed in **Supplementary Table 1**. Amplification of the sequence of interest was normalized to an endogenous reference control (*GAPDH*) and analyzed by the relative quantification method.

### Immunoblottings

Purified CD19^+^ CLL cells were lysed for 20 min on ice with lysis buffer supplemented with dithiothreitol and protease inhibitor cocktail (BioVision Cat# K269, Milpitas, CA, USA). Proteins (70 μg/lane) were electrophoresed on 4 to 20% of SDS-polyacrylamide gradient gels (Bio-Rad Laboratories Cat# 4561094, Hercules, CA, USA). Membranes were immunoblotted with primary antibodies listed in **Supplementary Table 2**. Then, membranes were incubated with a species-specific horseradish peroxidase (HRP)-conjugated secondary antibody (diluted 1:20,000) (Bethyl Cat# A120-101P, and Cat# A90-116) and developed using HRP conjugates Western Bright Sirius (Advasta, Menlo Park, CA, USA). Images were acquired by ChemiDoc XRS+ (Bio-Rad Laboratories) and analyzed using Image Lab Software v.3.0 (Bio-Rad Laboratories).

### Immunofluorescence

Expression levels of IDO1 protein in CD19^+^ CLL cells were also evaluated by intracytoplasmic immunofluorescence staining. CD19^+^ CLL cells were plated in RPMI-1640 medium + 10% FBS on coverslips in a 24-well plate and pretreated (or not) with INCB018424 for 1 h before the incubation with IFN-γ for 20 h. After stimulation, cells were fixed and permeabilized on coverslips. After washes, the primary IDO1 antibody was loaded on coverslips and incubated overnight at +4°C. The day after, CLL cells were washed and incubated with Alexa-Fluor 488 conjugated secondary antibody (Thermo Fisher Scientific Cat# A-11034) for 1 h at room temperature. Finally, DAPI Antifade ES (CytoCell Cat# DES500L, Cambridge, UK) was added to stain cell nuclei. Immunofluorescent images were visualized with EVOS™ M5000 Imaging System (Thermo Fisher Scientific).

### Flow Cytometry

A flow cytometric evaluation of IDO1 expression in CLL cells was assessed treating CD19^+^ cells isolated from CLL patients with IFN-γ for 24 h. Then, cells were fixed and permeabilized overnight. The following day, cells were washed and incubated with the anti-human IDO1-PE antibody (Cell Signaling Technology Cat# 10312) for 1 h on ice. For each sample, an isotype control was prepared in parallel.

### Sample Preparation for Metabolite Analysis

To assess the effect of IFN-γ on IDO1 activity, purified CD19^+^ CLL cells were resuspended in 0.5 ml of RPMI-1640 medium + 10% FBS and cultured at a density of 12 × 10^6^/well in 12-well plates. Cells were pretreated (or not) with INCB018424 1 µM for 1 h before the incubation with IFN-γ for 24 h. Similarly, 0.5 ml of conditioned media was collected from 5 × 10^6^ CLL cells 24 h post transfection with the IDO1 vector, or the corresponding empty vector. Conditioned media were collected by centrifugation at 2,000 × g for 15 min and stored at −80°C until assayed. Fifty µl of these supernatants was added with an equal volume of ice-cold 1 M perchloric acid (HClO_4_) fortified with a mix of the following stable isotope-labeled internal standard (final concentration 1 µM): L-kynurenine-d4 (Buchem BV, Apeldoorn, Netherlands) and L-tryptophan-d5 (Sigma-Aldrich). Samples were centrifuged (15,000 × g, 15 min), and the supernatants were collected and directly injected into LC-MS/MS.

### Liquid Chromatography and Chemicals

LC-MS/MS analyses were performed as previously described (31), with a few changes. The analyses were performed using an Agilent HP 1200 liquid chromatograph (Agilent, Santa Clara, CA, USA) consisting of a binary pump, an autosampler, and a thermostated column compartment. Chromatographic separations were carried out using a Discovery^®^ HS-F5-3 column (150 × 2.1 mm, 3 µm, Supelco Cat# 567503-U, Bellefonte, PA, USA) using 0.1% formic acid in water (solvent A) and acetonitrile (solvent B) as mobile phase. The HPLC analyses were performed using a linear elution profile of 15 min from 5% to 90% of acetonitrile (ACN). The column was then washed with 90% of ACN for 3.5 min followed by the equilibration of the column for 5 min with 5% ACN. The flow rate was 0.5 ml/min. The injection volume was 20 µl. An Agilent 6410 triple quadrupole-mass spectrometer with an electrospray ion source (ESI) operating in positive mode was used for detection. L-Tryptophan, HPLC-grade acetonitrile, and methanol were obtained from Sigma-Aldrich. Analytical grade formic acid, acetonitrile, and perchloric acid were obtained from Carlo Erba. Water was purified using the Milli-Q water purification system (Millipore).

### B-CLL Cell Transfection Strategy

CLL cells were transfected using either a plasmid vector or small interfering RNAs (siRNAs). The transfections were carried out in a Nucleofector instrument (Lonza, Basel, Switzerland) with the P3 primary cell solution kit using the program EO-117. Briefly, 5 × 10^6^ CD19^+^ CLL cells were transfected with 10 μg of IDO1 plasmid vector (Cat# HG11650-NF) or the corresponding empty vector pCMV3-Negative Control (Cat# CV020) (all from Sino Biological Inc., Shanghai, China). The expression of AHR was silenced using TriFECTa^®^ Kit DsiRNA Duplex (IDT Design ID# hs.Ri.AHR.13) at a concentration of 200 nM. Non-targeted negative control siRNA was used as negative control in all experiments. Transfected cells were subsequently plated in RPMI-1640 medium supplemented with 10% FBS and analyzed at indicated time points.

### CLL Cell Viability

The apoptotic cell death of CLL cells was analyzed using Annexin V-FITC and propidium iodide (PI) staining (Thermo Fisher Scientific Cat# BMS500FI/100). Viability was defined as the percentage of Annexin V-/PI- cells, while apoptosis was defined as the percentage of Annexin V+/PI- cells. Events were acquired using the BD Accuri™ C6 Plus Flow Cytometer System (Becton Dickinson) and then analyzed by FlowJo Software (Tree Star).

### Statistical Analyses

Data were analyzed using GraphPad Prism 6 (GraphPad Software) or R 3.6.3 software (The R Foundation for Statistical Computing). In some experiments, results were normalized on control (100%) (vehicle-treated samples). Normalization was performed by dividing the value of a particular treated sample to the value of the corresponding sample treated with vehicle. p values were calculated by Student paired t test, and repeated-measure two- or three-way ANOVA (*p < 0.05, **p < 0.01, ***p < 0.001). When applicable, experiments followed a complete factorial design with two or three experimental factors. Data from each experiment were analyzed with a repeated-measure approach. Firstly, a full model was estimated, including all pairwise and—if the case—higher-order interaction terms. When interaction terms were not statistically significant, they were removed from the models and only the main effects for each factor were estimated. Main effects were reported as the mean difference (MD) with 95% confidence intervals (CI). Repeated-measure analyses were carried out with linear mixed models with a random intercept for each individual and random slope terms for each factor (without interactions). Data are presented as mean and standard error of the mean (SEM) is depicted as error bars.

## Results

### IDO1 Is Expressed in CLL and Modulated by Stimuli That Mimic Tumor Microenvironment

**Figure 1:**
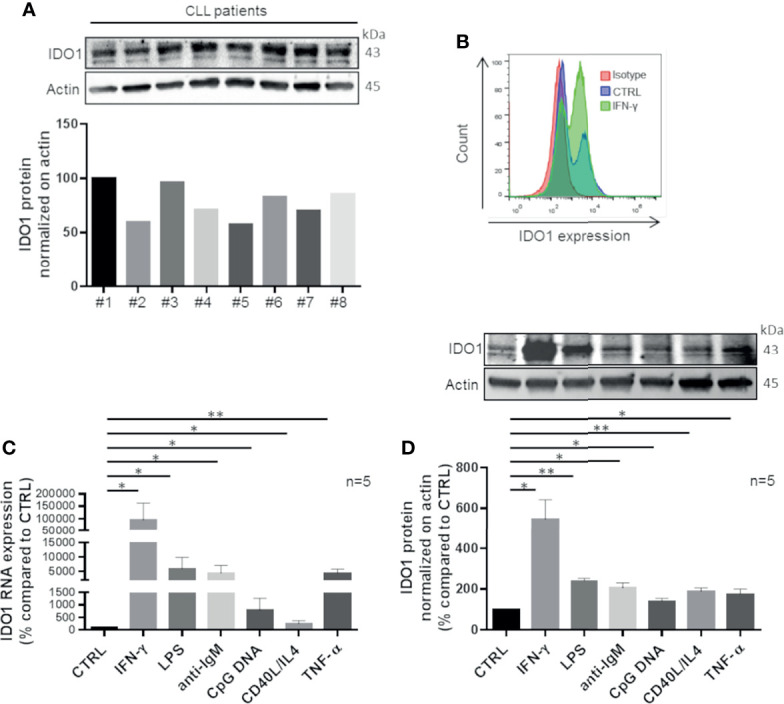
CLL cells up-regulate IDO1 in response to stimuli that mimic microenvironmental signals. **(A)** Purified leukemic CD19^+^ cells from CLL patients were serum starved for 1 h. Then the basal level of IDO1 was inspected by Western blot (n = 8) and illustrated by a bar diagram. **(B)** Flow cytometric histograms represent a high IDO1 level in CD19^+^ cells treated for 24 h with IFN-γ compared to the untreated control sample and isotype sample. **(C)** Bar diagram represents *IDO1* expression in CD19^+^ CLL measured by real-time PCR. Samples were treated for 4 h with one of microenvironment stimuli individually (Student paired t test, *p < 0.05, **p < 0.01; n = 5). **(D)** Immunoblots represent IDO1 protein induction after 24 h of stimulation with the abovementioned factors. Histograms below represent the densitometric quantifications (Student paired t test, *p < 0.05, **p < 0.01; n = 5).

Firstly, we characterized IDO1 expression in CLL. CD19^+^ cells were purified from peripheral blood of untreated patients and inspected for IDO1 mRNA expression, detecting a variable amount in all samples (data not shown). These data were confirmed analyzing the expression level of IDO1 protein as shown in **Figure 1A**. After exposure to IFN-γ, a known inducer of IDO1, CD19^+^ leukemic cells strongly upregulated IDO1 protein (**Figure 1B**). Given the pivotal role of TME in CLL progression and clonal evolution, we decided to evaluate the impact of microenvironmental signaling on IDO1 regulation in CLL. Purified CD19^+^ cells were treated with different stimuli to trigger the B cell receptor (BCR), toll-like receptor (TLR) 4, TLR 7-9, CD40, and TNF receptor, in order to mimic the CLL microenvironment. After 4 h of stimulation, we observed a significant increase in IDO1 mRNA level in samples treated with IFN-γ ( p = 0.037), with LPS (p = 0.028), with anti-IgM (p = 0.021), with CpG DNA (p = 0.032), with CD40L + IL-4 (p = 0.049), and with TNF-α (p = 0.003) (**Figure 1C**). Accordingly, we also detected significantly higher IDO1 protein in CLL cells following each stimulation if compared to controls after 24 h, as reported in **Figure 1D**.

### IFN-γ/Jak/STAT1 Pathway Regulates the Production of Enzymatically Active IDO1 in CLL

**Figure 2:**
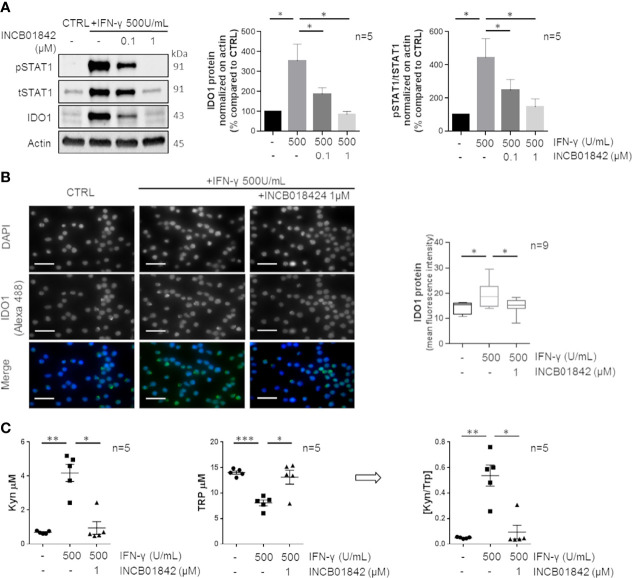
IDO1 protein induction and activity can be regulated by IFN-γ in CLL. **(A)** Purified CLL cells were pretreated with two doses (0.1–1 μM) of INCB018424 for 1 h prior to IFN-γ stimulation for 24 h. Immunoblots depict IDO1, tSTAT1, and pSTAT1 protein levels in a representative case. Histograms on the right represent the densitometric quantifications of IDO1 and pSTAT1/tSTAT1 in 5 CLL samples (Student paired t test, *p < 0.05; n = 5). **(B)** Immunofluorescence staining shows the ability of INCB018424 1 μM to reduce IDO1 expression induced by IFN-γ. Scale bar is 20 µm. Box plots summarize the fluorescent levels in DMSO, IFN-γ, or IFN-γ + INCB018424-treated CLL cells (Student paired t test, *p < 0.05; n = 9). **(C)** Conditioned media were collected after INCB018424 pretreatment (or not) and IFN-γ incubation for 24 h. Dot plots on the left represent the mean of the Kyn concentration measured by LC-MS/MS analytical technique in 5 separated experiments (Student paired t test, *p < 0.05, **p < 0.01; n = 5). The central dot plots represent the mean of the Trp concentration (Student paired t test, *p < 0.05, ***p < 0.001; n = 5). The resultant [Kyn]/[Trp] ratio calculated is depicted in the right dot plot (Student paired t test, *p < 0.05, **p < 0.01; n = 5).

We investigated whether overexpressed IDO1 was mediated by the Jak/STAT1 pathway and was enzymatically active. Firstly, we pretreated CLL cells with a JAK1/2 inhibitor, INCB018424, leading to the blockade of the intracellular signaling molecules downstream to IFN-γ. A dose escalation of INCB018424 (0.1–1 µM) significantly impaired the expression of IDO1 in a dose-dependent manner with a concomitant reduction of phosphorylated and total STAT1 protein levels induced by IFN-γ stimulation (**Figure 2A**, both p < 0.05). The reduction of IDO1 level by the most effective dose of INCB018424 (1 µM) was also confirmed by immunofluorescence (**Figure 2B**, p = 0.046). Because IDO1 activity can be indirectly estimated by determining the ratio between the amount of metabolites produced to the degraded substrate (32), we quantified the Kyn and Trp levels in the conditioned media of cultured CLL cells. Treatment of CD19^+^ cells with IFN-γ induced a significant production of Kyn (from 0.7 µM ± 0.1 µM to 4.2 µM ± 0.50 µM, p = 0.002) with a concomitant depletion of Trp (from 14.0 µM ± 0.3 µM to 8.1 µM ± 0.6 µM, p < 0.001) if compared to control. Again, treatment with INCB018424 1 µM significantly affected the production of Kyn (1.0 ± 0.4 µM, p = 0.018) (**Figure 2C**). As a result, the [Kyn]/[Trp] ratio calculated after IFN-γ stimulation increased considerably (from 0.05 ± 0.003 to 0.54 ± 0.083, p = 0.004) indicating the full activity of the IDO1 enzyme. A minimal IDO1 activity was observed in INCB018424-treated CLL cells, in which the [Kyn]/[Trp] ratio was significantly decreased (0.09 ± 0.054, p = 0.028) (**Figure 2C**). Overall, these findings showed that CLL cells respond to IFN-γ stimulation upregulating an active form of IDO1 protein, confirming that this enzyme is active and inducible in CLL.

### IDO1 Is Involved in Preserving CLL Survival

**Figure 3:**
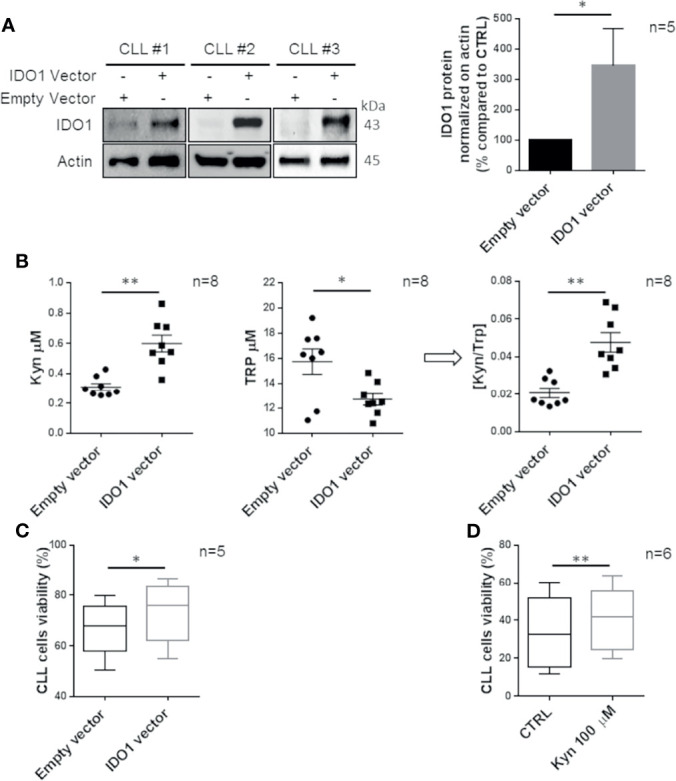
CLL cell survival is promoted by IDO1 overexpression. **(A)** CLL cells were transfected with an IDO1-expressing vector or an empty vector. After 24 h from transfection, the IDO1 protein level was measured by Western blot. Bar diagrams represent the densitometric quantifications of IDO1 (Student paired t test, *p < 0.05; n = 5). **(B)** Conditioned media were collected 24 h post transfection with the IDO1 vector or empty vector. Dot plots on the left represent the mean of the Kyn concentration measured by HPLC in 8 separated experiments (Student paired t test, **p < 0.01; n = 8). The central dot plots represent the mean of the Trp concentration (Student paired t test, *p < 0.05; n = 8). The resultant [Kyn]/[Trp] ratio calculated is depicted in the right dot plot (Student paired t test, **p < 0.01; n = 8). **(C)** Box plots represent the percentage of viable CLL cells after 24 h of transfection with IDO1-expressing vector or empty vector (Student paired t test, *p < 0.05; n = 5). **(D)** Box plots represent the percentage of CLL cell viability measured after 48 h of stimulation with Kyn 100 µM (Student paired t test, **p < 0.01; n = 6).

To specifically investigate the functional role of IDO1 in CLL cells, transfection of the IDO1 vector or empty vector as control was performed. We detected a significant IDO1 overexpression comparable to the induction previously obtained with IFN-γ stimulation (**Figure 3A**, p = 0.018). We used the LC-MS/MS procedure for Kyn and Trp dosage in conditioned media of IDO1-transfected CD19^+^ cells, confirming the increased activity of the overexpressed enzyme compared to the control (Kyn from 0.3 µM ± 0.02 µM to 0.6 µM ± 0.06 µM, p = 0.002; Trp from 15.7 µM ± 1.0 µM to 12.7 µM ± 0.5 µM, p = 0.028; [Kyn]/[Trp] ratio from 0.02 ± 0.003 to 0.05 ± 0.005, p = 0.005; **Figure 3B**). The genetic modulation of IDO1 determined an increase of CLL survival (from 66.9% ± 2.4% to 73.5% ± 2.7% of viable cells, p = 0.015) (**Figure 3C**). This result was also confirmed by measuring a decrease in the apoptotic rate after transfection with the IDO1 vector, as shown in **Supplementary Figure 1A**. Moreover, we treated purified CD19^+^ cells with exogenous Kyn to mimic the effect of IDO1 enzymatic action. As expected, Kyn promoted a significant increase in CLL cell viability (from 33.7% ± 3.8% to 41.1% ± 3.4%, p = 0.002) (**Figure 3D**) and a decrease in CLL cell apoptosis (**Supplementary Figure 1B**). These data confirmed the involvement of IDO1 and its derived metabolite in preserving CLL cell survival.

### AHR Promotes Cell Survival Modulating MCL1 in CLL Cells

**Figure 4:**
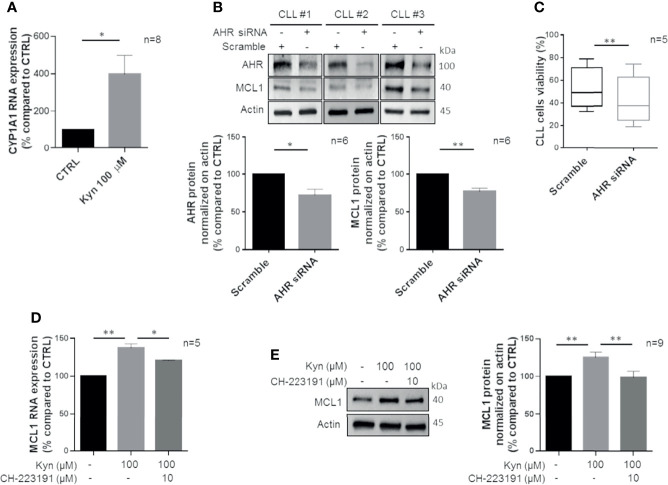
Kyn enhances MCL1 levels by activating AHR. **(A)** Bar diagram displays *CYP1A1* mRNA levels that were measured by real-time PCR in CD19^+^ cells treated with 100 μM Kyn, for 3 h (Student paired t test, *p < 0.05; n = 8). **(B)** Immunoblots show expression of AHR and MCL1 48 h after transfection with scramble or AHR siRNA in 3 representative CLL samples. Histograms show densitometric quantification of AHR and MCL1 protein level (Student paired t test, *p < 0.05, **p < 0.01; n = 6). **(C)** Box plots represent the percentage of viable CLL cells after 48 h from transfection with AHR siRNA or scramble (Student paired t test, **p < 0.01; n = 5). **(D)** *MCL1* mRNA levels were measured by real-time PCR in CLL cells pretreated for 90 min with 10 µM CH-223191 prior to addition of 100 µM Kyn. Histogram depicts the inhibition of MCL1 induction by CH-223191 (Student paired t test, *p < 0.05, **p < 0.01; n = 5). **(E)** Immunoblot depicts MCL1 protein levels in a representative case. On the right, histogram represents the densitometric quantifications of CLL samples (Student paired t test, **p < 0.01; n = 9).

Kyn is a potent endogenous activator of AHR (33), a ligand-controlled transcription factor that regulates enzymes metabolizing xenobiotic chemicals, such as cytochrome P450s (34). Therefore, we inspected if Kyn produced by CLL cells could act through an autocrine and/or paracrine loop on AHR. We observed that Kyn treatment is able to induce the transcription of *CYP1A1*, a known target of AHR (**Figure 4A**, p = 0.021). Then, we silenced AHR expression by siRNA transfection observing its significant impairment (**Figure 4B**, p = 0.02). Accordingly, CYP1A1 expression was decreased by AHR silencing (**Supplementary Figure 2A**). In this context, we detected a significant reduction of MCL1 expression in CLL cells consequent to the decreased AHR level (**Figure 4B**, p = 0.004). Of note, reduction of AHR determined a significant decrease in CLL cell viability (from 53.0% ± 4.1% to 42.3% ± 4.8%, p = 0.003) (**Figure 4C**). As a consequence, the apoptotic rate of AHR-silenced CLL cells was increased (**Supplementary Figure 2A**). Accordingly, we pretreated isolated CD19^+^ cells with CH-223191, an AHR antagonist, and then we analyzed the expression of MCL1. Inhibition of AHR was able to affect MCL1 expression induced by Kyn stimulation, both at transcriptional (from 137.1% ± 2.7% to 120.5% ± 0.4%, p = 0.028) (**Figure 4D**) and protein levels (from 128.6% ± 3.4% to 109.4% ± 4.2%, p = 0.002) (**Figure 4E**).

### MCL1 Induction by the IDO1/Kyn/AHR Axis Affects the Response to ABT-199 in CLL

**Figure 5:**
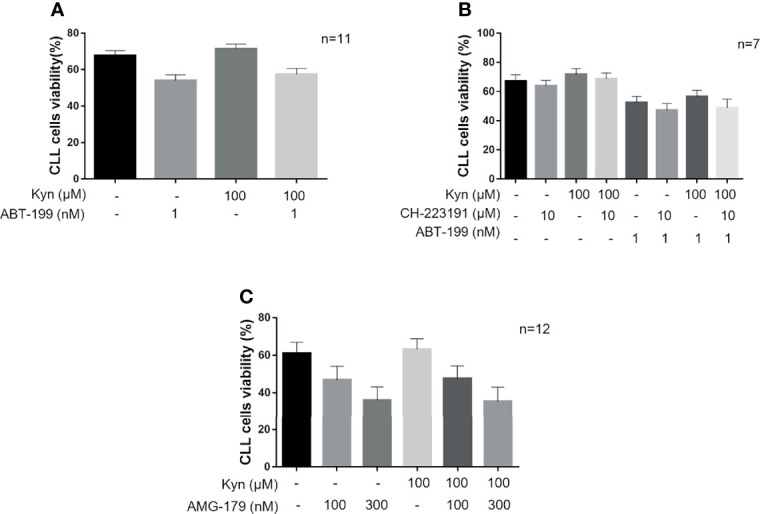
The activity of IDO1/Kyn/AHR axis impairs CLL cells response to ABT-199. **(A)** Histograms show comparison on viability rate of CLL cells treated with 1 nM ABT-199 for 5 h after overnight stimulation with 100 µM Kyn (repeated-measure two-way ANOVA with Kyn and CH-223191 treatment groups as the two factors; n = 11). **(B)** CLL cells were pretreated for 90 min with 10 µM CH-223191 and cultured overnight with Kyn. Then, 1 nM ABT-199 was added to culture for 5 h prior to cell viability assessment. Histograms illustrate the synergistic effect of CH-223191 with ABT-199 in CLL cells (repeated-measure three-way ANOVA with Kyn, ABT-199, and CH-223191 treatment groups as the three factors; n = 7). **(C)** CD19+ cells were cultured overnight with 100 µM Kyn, and then 100 or 300 nM AMG-176 was added to culture for 5 h. Cell viability was measured and depicted in histograms (repeated-measure two-way ANOVA with Kyn and AMG-176 treatment groups as the two factors; n = 12).

A high expression of B-cell lymphoma-extra large (BCL-xL) and MCL1 has reported to contribute to reduced response to ABT-199 in CLL (35). Therefore, to evaluate the effect of IDO1-induced survival on CLL cell sensitivity to ABT-199 treatment, we treated CD19^+^ cells with Kyn, and then we added or not ABT-199 1 nM. We found that the killing effect of the BCL2 inhibitor was impaired in CLL because of the Kyn-mediated survival signal. Indeed, Kyn pre-incubation improved the percentage of viable cells in both settings, with or without ABT-199 treatment, from 54.7% ± 1.6% to 58.4% ± 1.6% and from 68.0% ± 1.4% to 71.8% ± 1.3%, respectively (**Figure 5A**). The interaction effect was not significant. The main effects were MD = 3.76, [95% CI = 2.12; 5.40], p = 0.001, for Kyn treatment and MD = -13.32, [95% CI = -20.73; -5.92], p = 0.006, for ABT-199 treatment. Given the important role of the IDO1/Kyn/AHR axis in the induction of MCL1 documented above, we pretreated CD19^+^ cells with CH-223191 to block the signaling downstream to Kyn prior to ABT-199 1-nM addition. AHR inhibition promoted apoptotic cell death because CH-22311 showed an additive effect with ABT-199 in CLL (cell viability from 56.6% ± 2.1% to 48.8% ± 3.0%) (**Figure 5B**) as deduced from the main effects obtained for the three treatments (MD = 3.80, [95% CI = 0.88; 6.73], p = 0.043; MD = -4.81, [95% CI = -7.00; -2.62], p = 0.003; MD = -16.63, [95% CI = -26.79; -6.46], p = 0.019 for Kyn, Ch-223291 and ABT-199 treatments, respectively). Since inhibition of MCL1 is effective in CLL cells and the MCL1 antagonist, AMG-176, has been demonstrated to induce apoptosis in the CLL setting (36), we wanted to evaluate if AMG-176 could impair Kyn-mediated survival of CLL cells. Hence, isolated CD19^+^ cells were pre-incubated with Kyn and then two doses of AMG-176 (100–300 nM) were tested. After 5 h of culture with AMG-176, we detected in all our conditions a significant induction of apoptosis (**Figure 5C**). Since the interaction effect was not significant, the main effects were MD = -1.238, [95% CI = -2.966; 0.4911], p = 0.14, and MD = 15.45, [95% CI = 10.76; 20.15], p < 0.001, for Kyn and AMG-176 100 nM treatment; MD = -0.7250, [95% CI = -2.215; 0.7651], p = 0.31, and MD = 29.68, [95% CI = 21.29; 38.06], p < 0.001, for Kyn and AMG-176 300 nM treatment, respectively. However, directly inhibiting MCL1, AMG-176 completely nullified the survival effect of Kyn, as no difference in cell viability was found in the comparison between samples treated with Kyn and AMG-176 or the MCL1 inhibitor alone. Summarizing, these data showed that the IDO1/Kyn/AHR axis induces survival in CLL leukemic cell through the maintenance of high levels of MCL1.

## Discussion

IDO1 is an intracellular enzyme that initiates the first and rate-limiting step of Trp breakdown along the kynurenine pathway. In different tumors, IDO1 is constitutively expressed by the tumor cells themselves and also by tumor-associated cells, such as dendritic cells or endothelial cells (17).

Here, we demonstrated for the first time that CLL cells express an active and functional form of IDO1 enzyme and that microenvironmental stimuli, as inflammatory and pro-survival factors, are able to strongly induce a positive modulation of IDO1 expression. In line with previous literature, IFN-γ strongly induces IDO1 expression through the Jak/STAT1 signaling pathway in CLL. The kynurenine-to-tryptophan ratio is frequently used to measure or reflect the activity of IDO1. The elevated plasma level of the [Kyn]/[Trp] ratio was detected in patients diagnosed with various solid tumors but also in CLL cases and often correlate with poor prognosis (18–24, 26). High levels of Kyn concomitantly with consumption of Trp were detected in conditioned media collected from CD19^+^ CLL cells stimulated with IFN-γ, confirming that the overexpressed IDO1 enzyme was active in CLL cells. Accordingly, we found that blocking IFN-γ signaling *via* INCB018424 interferes with IDO1 induction, STAT1 phosphorylation, and Kyn production. Overall, our findings suggested that IDO1 may be abnormally produced and activated in leukemic cells in response to signals known to be present inside the tissue microenvironment.

Nowadays, attention has been focused on the effects of IDO1, because in addition to immune regulation, activation of the IDO1/Kyn/AHR axis affects tumor cell viability, proliferation, and apoptosis *in vitro*. It was demonstrated that blockade of IDO1 activity in cancer cells could reduce β-catenin activation and inhibit cell proliferation (37). IDO1 silencing in melanoma cells inhibited cancer cell proliferation and induced cell apoptosis (38). We argued whether the IDO1 pathway may trigger survival stimuli on CLL cells by establishing an autocrine loop and/or by acting throughout the microenvironment. We observed an enhanced resistance to spontaneous apoptosis in CLL cells when IDO1 expression was forced with the plasmid vector. This pro-survival influence induced by IDO1 is also confirmed by treating CLL cells with Kyn, the main metabolite of the IDO1 enzyme. Accordingly, we demonstrated that Kyn activates AHR transcriptional activity in CLL cells. AHR is involved in the formation of tumors as its activation enhanced clonogenic survival and motility of tumor cells (39, 40) and as transgenic mice with a constitutively active AHR spontaneously develop tumors (41). As a consequence, we argued whether AHR may have a role in CLL survival and maintenance. Although the study of Gonder and colleagues showed that the conditional knockout of AHR in CD19^+^ B cells of the Eµ-TCL1 mouse model seems to have no impact on CLL progression or survival *in vivo* (42), we demonstrated that AHR silencing on CLL cells significantly increased apoptosis of CD19^+^ clones *in vitro*. These conflicting findings keep open the debate about AHR’s active involvement in CLL leukemogenesis and maintenance, which requires further investigation.

The BCL2 family proteins are well-known modulators of apoptosis. B-cell malignancies, such as CLL and follicular lymphomas, are functionally dependent on BCL2 for survival (43). Surprisingly, CLL cells treated with AHR agonist Kyn showed increased MCL1 transcript and protein. Conversely, we found that blocking AHR *via* CH-223191 or by AHR silencing abrogates MCL1 expression. Our data suggest that AHR blocking interferes with the survival of CLL cells by limiting the expression of MCL1. Here, we also evaluated whether IDO1/Kyn/AHR signaling may determine protection against drug-induced apoptosis as reported in solid tumors (44, 45). Mature B-cells, both normal and leukemic (CLL), are highly sensitive to BCL2 inhibition by ABT-199, which induces BAX/BAK-mediated apoptosis triggered mainly by BIM (46), but ABT-199 is much less effective against CLL cells that have received survival signals from the microenvironment (47). Sustained engagement of the BCR induces MCL1 (48), and high levels of MCL1 have been shown to protect CLL, other hematological malignancies, and certain solid tumors from ABT-199 (47). We found that Kyn-treated CLL cells are less affected by the pro-apoptotic effect of ABT-199. AHR blockade by the CH-223191 antagonist inhibited the Kyn-mediated protection against ABT-199-induced apoptosis. Moreover, at low concentrations of ABT-199, CH-223191 showed synergistic or additive cytotoxicity to CLL lymphocytes. Finally, we wanted to confirm that the IDO1/Kyn/AHR axis improves CLL survival through MCL1 induction, targeting MCL1 itself. AMG-176 is a selective and direct antagonist of MCL1, which has shown efficacy in several hematological malignancies, including CLL (36). As expected, CD19^+^ cells rapidly went into apoptosis after AMG-176 treatment even if treated with Kyn. Indeed, AMG-176 completely neutralized the survival effect of the IDO1/Kyn/AHR pathway in CLL. In this scenario, the IDO1 pathway activated in CLL cells by stimuli from other cell types, such as endothelial cells, NLCs, MDSCs, and T cells inside microenvironmental niches, could interfere with the effect of novel targeted drugs currently used for CLL patients’ management. The results support the notion that the activation status of the IDO1/Kyn/AHR axis may be of relevance in CLL clinical outcome.

Several issues concerning the role of IDO1 in CLL remain to be explored. First, multiple mechanisms at both transcriptional (i.e., Bridging integrator 1, Bin1) and posttranslational levels (i.e., suppressor of cytokine signaling 3, SOCS3) may be implicated in the abnormal levels of IDO1. Second, the mechanisms underlying AHR induction of MCL1 are not fully elucidated. Upon ligand binding, AHR translocates to the nucleus and the formation of AHR-AHR nuclear translocator (ARNT) heterodimer leads to the transcription of dioxin-responsive element-containing genes (49). In addition to the canonical pathway characterized by AHR nuclear translocation through which AHR regulates target gene expression, AHR influences many biological processes through non-genomic signaling mechanisms (45). Further studies are needed to clarify the molecular cascade that links IDO1 activity to MCL1 expression through not only Kyn and AHR. On this line, we have planned to confirm our findings in an adoptive transfer mouse model of CLL. An *in vivo* study will allow (i) to evaluate the effects of the IDO1/Kyn/AHR axis on the complexity of the leukemic microenvironment; (ii) to consider the impact of the known deregulation of nicotinate/nicotinamide pathways and purine metabolism by the action of IDO1 (50); and (iii) to analyze the effects of IDO1 and AHR inhibitors on the leukemic population evaluating the disease dynamics (insurgence, aggressiveness, progression and remission).

In conclusion, our data show for the first time that CLL cells express an active IDO1 enzyme that produces high levels of Kyn consuming Trp *via* the kynurenine pathway. The results also demonstrate that the IDO1/Kyn/AHR axis plays a role in survival and drug resistance of leukemic cells. The observed ability of the AHR selective antagonist to interfere with protective signals of CLL cells may be explored, both experimentally and clinically, as a possible novel therapeutic approach in CLL.

## Data Availability Statement

The original contributions presented in the study are included in the article/**Supplementary Material**. Further inquiries can be directed to the corresponding author.

## Ethics Statement

The studies involving human participants were reviewed and approved by 1003/2018/SPER/AOUMO – IDO in CLL-”Indoleamina-2,3-diossigenasi come mediatore della tolleranza immunitaria nella leucemia linfatica cronica – IDO”. The patients/participants provided their written informed consent to participate in this study.

## Author Contributions

RMaf and RMar conceived and coordinated the research and interpreted the results. CA performed the *in vitro* experiments, performed the statistical analyses, interpreted the results, and wrote the manuscript. SF acquired and analyzed the flow cytometric data. NM performed molecular analyses and contributed to Western blot analyses. SA performed LC-MS/MS analyses. MM and GL provided patient samples and clinical data. SM managed the biological samples. LP, ML, RMaf, and RMar revised and approved the final version of the manuscript. All authors contributed to the article and approved the submitted version.

## Funding

This work was supported by Associazione Italiana per la Ricerca sul Cancro (IG21436 RMar) and Progetto Dipartimenti di Eccellenza 2018–2022. SF was supported by an annual fellowship from Fondazione Umberto Veronesi, Italy.

## Conflict of Interest

The authors declare that the research was conducted in the absence of any commercial or financial relationships that could be construed as a potential conflict of interest.

## Publisher’s Note

All claims expressed in this article are solely those of the authors and do not necessarily represent those of their affiliated organizations, or those of the publisher, the editors and the reviewers. Any product that may be evaluated in this article, or claim that may be made by its manufacturer, is not guaranteed or endorsed by the publisher.

## Supplementary Material

Supplementary Figure 1 IDO1 overexpression and Kyn treatment reduce apoptosis of CLL cells. **(A)** Box plots represent the percentage of apoptotic CLL cells after 24 h of transfection with IDO1-expressing vector or empty vector (Student paired t test, *p < 0.05; n = 5). **(B)** Apoptotic CLL cells measured after 48 h of stimulation with Kyn 100 µM are depicted in box plots (Student paired t test, **p < 0.01; n = 6).

Supplementary Figure 2 Silencing of AHR in CLL cells reduces CYP1A1 protein expression and enhances apoptosis. **(A)** Immunoblots show expression of CYP1A1 48 h after transfection with scramble or AHR siRNA in 3 representative CLL samples. Histograms show densitometric quantification of CYP1A1 protein level (Student paired t test, *p < 0.05; n = 6). **(B)** Box plots represent the percentage of apoptotic CLL cells after 48 h from transfection with AHR siRNA or scramble (Student paired t test, ***p < 0.001; n = 5).

Supplementary Table 1 List of primers used for Real Time PCR

Supplementary Table 2 List of primary antibodies used in this study
