# Supplementary figures and images for: Indoleamine 2, 3-Dioxygenase 1 Mediates Survival Signals in Chronic Lymphocytic Leukemia via Kynurenine/Aryl Hydrocarbon Receptor-Mediated MCL1 Modulation
Source: Front Immunol. 2022 Mar 18;13:832263. doi: 10.3389/fimmu.2022.832263 (PMC8971515; doi:10.3389/fimmu.2022.832263)

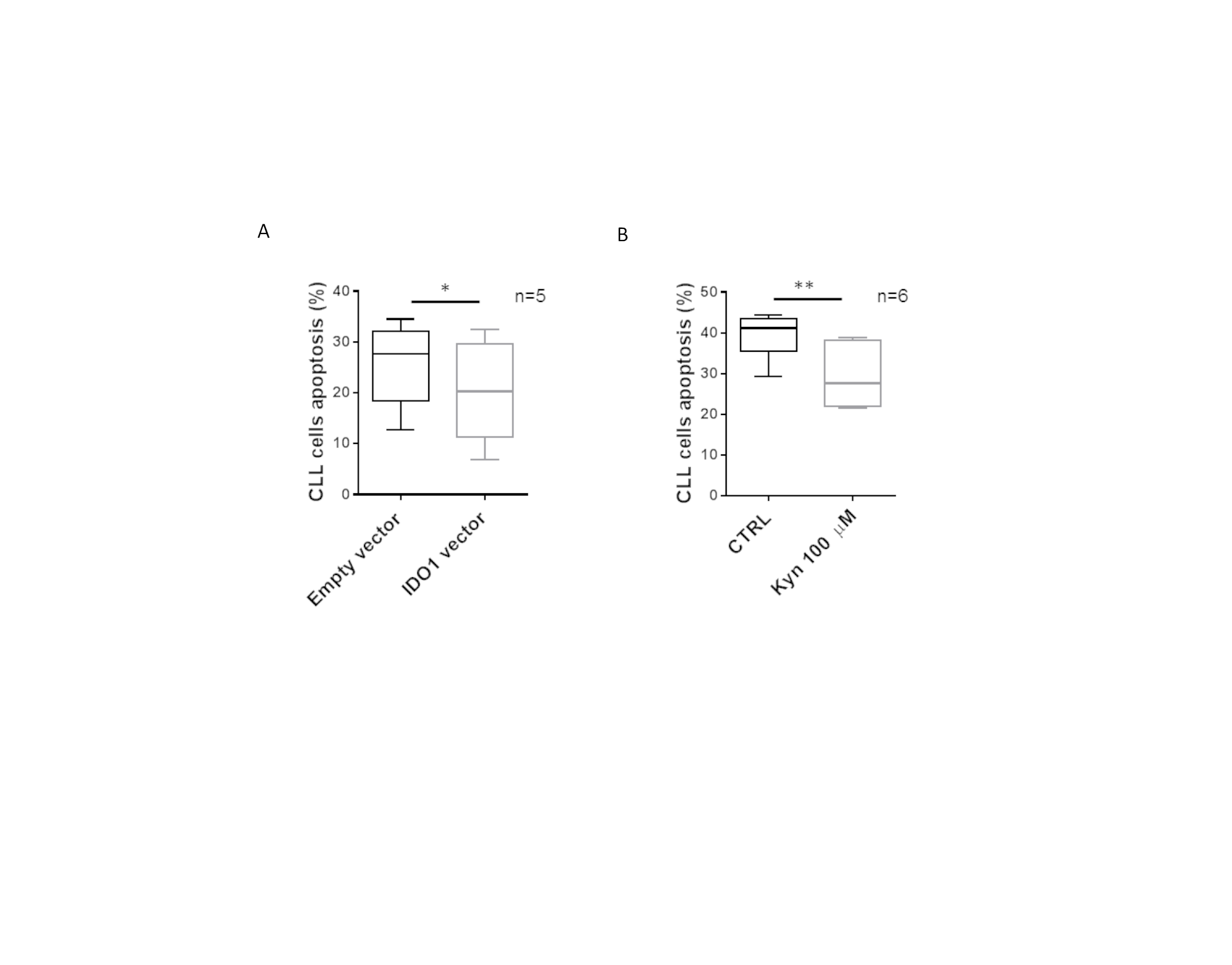

Supplement: Supplementary Figure 1 — IDO1 overexpression and Kyn treatment reduce apoptosis of CLL cells. (A) Box plots represent the percentage of apoptotic CLL cells after 24 h of transfection with IDO1-expressing vector or empty vector (Student paired t test, *p < 0.05; n = 5). (B) Apoptotic CLL cells measured after 48 h of stimulation with Kyn 100 µM are depicted in box plots (Student paired t test, **p < 0.01; n = 6). [file Image_1.tiff]

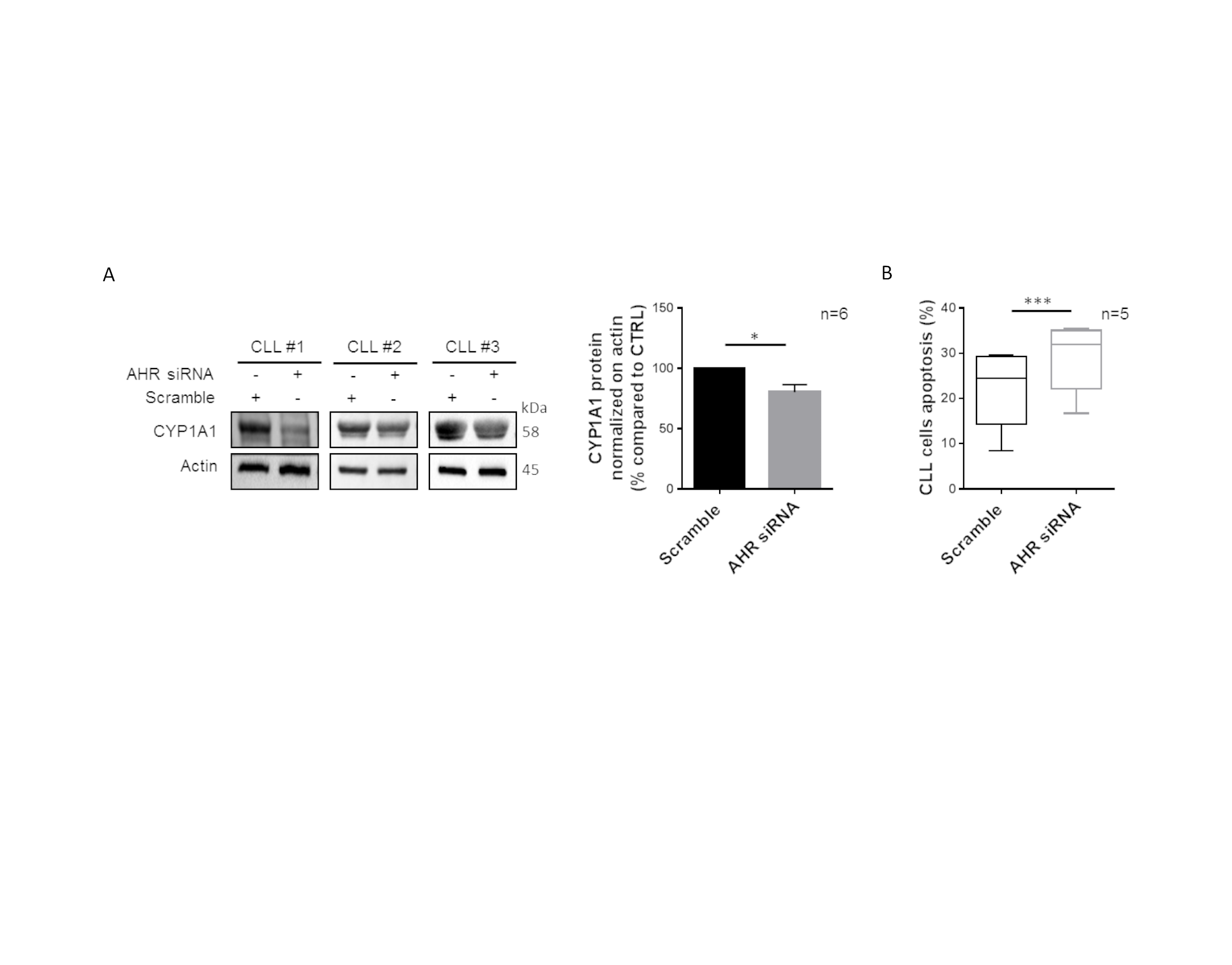

Supplement: Supplementary Figure 2 — Silencing of AHR in CLL cells reduces CYP1A1 protein expression and enhances apoptosis. (A) Immunoblots show expression of CYP1A1 48 h after transfection with scramble or AHR siRNA in 3 representative CLL samples. Histograms show densitometric quantification of CYP1A1 protein level (Student paired t test, *p < 0.05; n = 6). (B) Box plots represent the percentage of apoptotic CLL cells after 48 h from transfection with AHR siRNA or scramble (Student paired t test, ***p < 0.001; n = 5). [file Image_2.tiff]
