# Supplementary material for: Indoleamine 2, 3-Dioxygenase 1 Mediates Survival Signals in Chronic Lymphocytic Leukemia via Kynurenine/Aryl Hydrocarbon Receptor-Mediated MCL1 Modulation
Source: Front Immunol. 2022 Mar 18;13:832263. doi: 10.3389/fimmu.2022.832263 (PMC8971515; doi:10.3389/fimmu.2022.832263)
Supplement: Supplementary Table 1 — List of primers used for Real Time PCR [file Table_1.pdf]

Supplementary Table 1: List of primers used for Real Time PCR

Gene	Primers
IDO1	**Forward:** 5'- GTGTTTCACCAAATCCACGAT -3'
	**Reverse:** 5'- CTGATAGCTGGGGGTTGC -3'
CYP1A1	**Forward:** 5'- ACCTTCCCTGATCCTTGTGA -3'
	**Reverse:** 5'- GATCTTGGAGGTGGCTGCT -3'
MCL1	**Forward:** 5'- AAGCCAATGGGCAGGTCT -3'
	**Reverse:** 5'- TGTCCAGTTTCCGAAGCAT -3'
GAPDH	**Forward:** 5'- AGCCACATCGCTCAGACAC -3'
	**Reverse:** 5'- GCCCAATACGACCAAATCC -3'
