# Supplementary material for: Indoleamine 2, 3-Dioxygenase 1 Mediates Survival Signals in Chronic Lymphocytic Leukemia via Kynurenine/Aryl Hydrocarbon Receptor-Mediated MCL1 Modulation
Source: Front Immunol. 2022 Mar 18;13:832263. doi: 10.3389/fimmu.2022.832263 (PMC8971515; doi:10.3389/fimmu.2022.832263)
Supplement: Supplementary Table 2 — List of primary antibodies used in this study [file Table_2.pdf]

Supplementary Table 2: List of primary antibodies used in this study

Antigen	Clone	Dilution
IDO1	(Cell Signaling Technology Cat# 86630)	1:1 000
pSTAT1	(ECM Biosciences Cat# SM1351)	1:1 000
tSTAT1	(Cell Signaling Technology Cat# 9176)	1:1 000
MCL1	(Cell Signaling Technology Cat# 94296)	1:1 000
AHR	(Cell Signaling Technology Cat# 83200)	1:1 000
CYP1A1	(Bio-Rad Laboratories Cat# VPA00711)	1:1 000
Actin	(Abcam Cat# ab6276)	1:80 000
